# Supplementary material for: Screening for prevalence of current TB disease and latent TB infection in type 2 diabetes mellitus patients attending a diabetic clinic in an Indian tertiary care hospital
Source: PLoS One. 2020 Jun 5;15(6):e0233385. doi: 10.1371/journal.pone.0233385 (PMC7274437; doi:10.1371/journal.pone.0233385)
Supplement: Attachment — Submitted filename: Response to Reviewers.docx [file pone.0233385.s001.docx]

Response to reviewers:

|  | Reviewer comment | Author response | Changes made in manuscript |
| --- | --- | --- | --- |
| Rev 1 |  |  |  |
| 1 | Introduction: explain better the burden of TB in your country and compare it with global burden both TB and DM and why they are linked | The suggested changes made. | Pizzol D, Tuberculosis and diabetes: current state and future perspectives. Trop Med Int Health. 2016 Jun;21(6):694-702) |
| 2 | Methods: well wrote | Thank you |  |
| 3 | Results: 1000 patients are a very important sample. | Thank you |  |
| 4a | data from other low income setting | The suggested reference included. | Pizzol D et al. Prevalence of diabetes mellitus in newly diagnosed pulmonary tuberculosis in Beira, Mozambique.Afr Health Sci. 2017 Sep;17(3):773-779 |
| 4b | How is important treat and not only test diabetes | The suggested reference included. | Di Gennaro f et al Diabetes in active tuberculosis in low-income countries: to test or to take care?Lancet Glob Health. 2019 Jun;7(6):e707 |
| 4c | How diabetes is a predictors failure of TB | This study is a cross sectional study and does not look at TB outcomes | Pizzol D et alPredictors of therapy failure in newly diagnosed pulmonary tuberculosis cases in Beira, Mozambique.BMC Res Notes. 2018 Feb 5;11(1):99 |
| 4d | And how frugal technology as ultrasound can help TB diagnosis in poor setting | The suggestion isaccepted. We have cited an article that addresses this issue. | Bobbio F et al Focused ultrasound to diagnose HIV-associated tuberculosis (FASH) in the extremely resource-limited setting of South Sudan: a cross-sectional study.BMJ Open. 2019 Apr 2;9(4):e027179.and Di Gennaro F et al Potential Diagnostic Properties of Chest Ultrasound in Thoracic Tuberculosis-A Systematic Review.Int J Environ Res Public Health. 2018 Oct 12;15(10) |
| Rev 2 | No innovation, just simplly collected and calculated the data. The authors need to described and analyzed the data in manuscript in more details. | This is a prospective study and required the investigator to screen the subjects in the in a Diabetic clinic. Such studies are rare. |  |
| 3 | Line 182 It should be “4” rather than “3” categories | Thanks for pointing out | Correction made |
|  | Line 207 Have you got the BCG vaccination history of those patients? If a patient was BCG vaccinated, a positive TST test may not mean he/she has LBTI. A Quantiferon blood test should be considered to confirm it is a real LBTI. | That is correct. However, in this study Quantiferon test was not done due to non-availability locally. | We have mentioned this as a drawback and made the edits. |
|  | Line 250 It should be Table 2 not Table 3. | Thanks for pointingout | Corrections made |
|  | Line 250-251 What is normal BMI? The authors should give a definition. It seems that the authors considered BMI>23 as normal, please justify it by providing references. | BMI>23 is taken as abnormal in Indians. | Reference cited |
|  | Line 254-255 It says, “There was no difference between the groups with regards to HbA1c, fasting and post prandial plasma glucose”. I cannot find data of fasting and post prandial plasma glucose in Table2, please include. | Data is available on the table. | It has been moved up on the table and renamed as per the statement in the results |
|  | Line 258 should be “59%” not “9%” | Thanks, forpointing out | Corrections made |
|  | Line 260 delete “DM” | “DM” is required as prefix to treatment. | Not changed |
